# Human mesenchymal amniotic fluid stem cells reveal an unexpected neuronal potential differentiating into functional spinal motor neurons

**DOI:** 10.3389/fcell.2022.936990

**Published:** 2022-07-22

**Authors:** Giulia Gaggi, Andrea Di Credico, Simone Guarnieri, Maria Addolorata Mariggiò, Angela Di Baldassarre, Barbara Ghinassi

**Affiliations:** ^1^ Department of Medicine and Sciences of Aging, Chieti, Italy; ^2^ Reprogramming and Cell Differentiation Lab, Center for Advanced Studies and Technology (CAST), Chieti, Italy; ^3^ Department of Neuroscience, Imaging and Clinical Sciences, Chieti, Italy; ^4^ Functional Biotechnologies Lab, Center for Advanced Studies and Technology (CAST), University “G. d’Annunzio” of Chieti-Pescara, Chieti, Italy

**Keywords:** amniotic fluid stem cells (AFSC), mesenchimal cells, motoneuron (MN), regenerative medicine, perinatal stem cells

## Abstract

Human amniotic fluids stem cells (hAFSCs) can be easily isolated from the amniotic fluid during routinely scheduled amniocentesis. Unlike hiPSCs or hESC, they are neither tumorigenic nor immunogenic and their use does not rise ethical or safety issues: for these reasons they may represent a good candidate for the regenerative medicine. hAFSCs are generally considered multipotent and committed towards the mesodermal lineages; however, they express many pluripotent markers and share some epigenetic features with hiPSCs. Hence, we hypothesized that hAFSCs may overcome their mesodermal commitment differentiating into to ectodermal lineages. Here we demonstrated that by the sequential exposure to specific factors, hAFSCs can give rise to spinal motor neurons (MNs), as evidenced by the gradual gene and protein upregulation of early and late MN markers (PAX6, ISL1, HB9, NF-L, vAChT). When co-cultured with myotubes, hAFSCs-derived MNs were able to create functional neuromuscular junctions that induced robust skeletal muscle contractions. These data demonstrated the hAFSCs are not restricted to mesodermal commitment and can generate functional MNs thus outlining an ethically acceptable strategy for the study and treatment of the neurodegenerative diseases.

## Introduction

The irreversible and progressive degeneration of MNs leads to the onset of a group of neurological disorders called motor neuron diseases (MNDs), that cause weakness, progressive paralysis, and death. The survival of patients is about 3 years after the diagnosis and the available pharmacological treatments cannot block the progression of the disease (McDermott and Shaw, 2008; Foster and Salajegheh, 2019). To date the genetic causes and the molecular mechanisms involved in the MNDs onset and progression are still poorly understood; for this reason, the development of *in vitro* MNDs models is a very important goal, also for testing new and more effective drugs. Nowadays the human (h) MNs are obtained with high efficiency from embryonic stem cells (ESCs) and induced pluripotent stem cells (iPSCs); however, the use of ESCs is forbidden in many countries due to ethical issues, while the generation and the maintenance of hiPSCs require very specialized skills and great costs (Bilic and Belmonte, 2012; Di Baldassarre et al., 2018a).

In the past recent years, the biomedical research has been focused on a new class of stem cells, the perinatal stem cells: these represent a cellular population that can be easily isolated during routine scheduled amniocentesis or from the discarded fetal annexes after delivery without ethical concerns; moreover, they are nor tumorigenic neither immunogenic, and do not rise safety issues (Gaggi et al., 2019; Torre and Flores, 2020). In particular, amniotic fluid includes a heterogeneous cell population, usually derived from the embryo, from which is possible to isolate undifferentiated amniotic fluid stem cells (hAFSCs) by using specific selection process and/or permissive growth conditions. These cells proliferate rapidly and can be kept in culture for many passages without changes in gene and protein expression (Antonucci et al., 2014). hAFSCs are generally considered multipotent and committed towards the mesodermal lineages (Arnhold et al., 2011; Abdulrazzak et al., 2013; Loukogeorgakis and De Coppi, 2017; Di Baldassarre et al., 2018b). However, it has been shown that they express also many pluripotent markers such as NANOG, OCT4, SOX2, REX1 (Antonucci et al., 2014; Gaggi et al., 2019; Gaggi et al., 2020b) and have some epigenetic features similar to the ones of hiPSCs (Gaggi et al., 2020b). Hence, we hypothesised that hAFSCs may overcome the mesodermal commitment differentiating into ectodermal lineages. Aim of this study is to analyse whether hAFSCs can give efficiently rise to a MN progeny.

## Materials and methods

### Cell culture

Amniotic fluid samples were obtained from healthy women undergoing scheduled amniocentesis for prenatal diagnosis at 16–17 weeks of pregnancy after written informed consent, in accordance with the Declaration of Helsinki. The study was approved by the local ethics committee and all experiments were performed in accordance with relevant guidelines and regulations. To avoid the possible contamination with maternal cells, only normal diploid male karyotypes were included in the study. hAFSCs were isolated as previously described (Di Baldassarre et al., 2018b) Briefly, two or 3 ml of AF were obtained from 3 patients and cells were isolated by centrifugation at 1,200 rpm for 10 min at room temperature (RT). Adherent cells were cultured in Iscove’s modified Dulbecco’s medium (IMDM, Thermo Fisher Scientific, Waltham, MA, United States), supplemented with 20% fetal bovine serum (FBS, Thermo Fisher Scientific, United States), 100 U/mL penicillin/streptomycin, 2 mM L-glutamine, (all from Sigma-Aldrich, Saint Louis, MO, United States), and 5 ng/ml basic fibroblast growth factor (bFGF, Thermo Fisher Scientific, United States), and incubated at 37°C with 5% humidified CO_2_. The first change of the culture medium was performed after 1 week, and non-adherent cells were removed. Then the medium was changed when the cultures had reached 70%–80% confluence; all the experiments were performed between the 3 and 4 passages.

C2C12 cell line was purchased from ATCC (Mannassan, VA, United States) and cultured in Dulbecco’s Modified Eagle Medium (DMEM, Thermo Fisher Scientific, United States) 10% FBS, supplemented with 1% penicillin/streptomycin and 2 mM L-glutamine. For myotube differentiation, C2C12 at 90% of confluence were cultured in low serum (2% FBS) for 5–6 days.

### Flow cytometry

Phenotypical characterization of hAFSCs was performed between passages 2 and 4, as previously described (Di Baldassarre et al., 2018b). Briefly, cells were treated with the FIX & PERM^®^ Kit (Thermo Fisher Scientific) and then incubated for 1 h at RT with human anti-CD90-Alexa fluor 488-conjugated, CD34-Alexa fluor 488-conjugated, CD45-Alexa fluor 488-conjugated, SSEA4-Alexa fluor conjugated, OCT4-Alexa fluor 488-conjugated, Tra-1–60-Alexa fluor 488-conjugated, C-KIT-PE-conjugated, CD105-FITC-conjugated, NANOG-Alexa fluor 647-conjugated, SOX2-Alexa fluor 488-conjugated (All from Becton Dickinson, Franklin Lakes, NJ, United States) diluted 1:50–1:100 according to the manufacturer’s instructions. Cells incubated with isotypes (all from Becton Dickinson) were used as negative controls. Cytometric analysis was performed with a Cytoflex cytometer (Beckman Coulter Pasadenia, CA, United States), and data were analyzed with CytExpert Acquisition and Analysis Software (Beckman Coulter).

### Motor neuron differentiation

For motor neuron differentiation, cells were seeded at high density (5.2 × 10^4^ cell/cm^2^) on Matrigel (Corning, Flintshire, United Kingdom)-coated plates in IMDM 20% FBS and sequentially exposed to SB431542 hydrate (10 μM, Sigma-Aldrich, United States), LDN193189 (100 nM Stemgent, Cambridge, MA, United States), CHIR-99021 (3 μM, Tocris Bioscience, Bristol, United Kingdom), basic FGF (10 ng/ml Thermo Fisher Scientific, United States), ascorbic acid (10 µM Sigma-Aldrich, United States), RA (100 nM Sigma-Aldrich, United States), Smoothened agonist (SAG) (500 nM, Sigma-Aldrich, United States), DAPT (10 μM, Tocris Bioscience, Bristol, United Kingdom), BDNF (10 ng/ml, R&D system, United States), GDNF (20 ng/ml, R&D system, United States), CTNF (10 ng/mL R&D system, United States) β-mercaptoethanol (βMOH) (25 µM Sigma-Aldrich, United States), Forskolin (10 µM STEMCELL Technologies, Vancouver, British Columbia, Canada), IBMX (100 μM, R&D system, United States).

**FIGURE 1:**
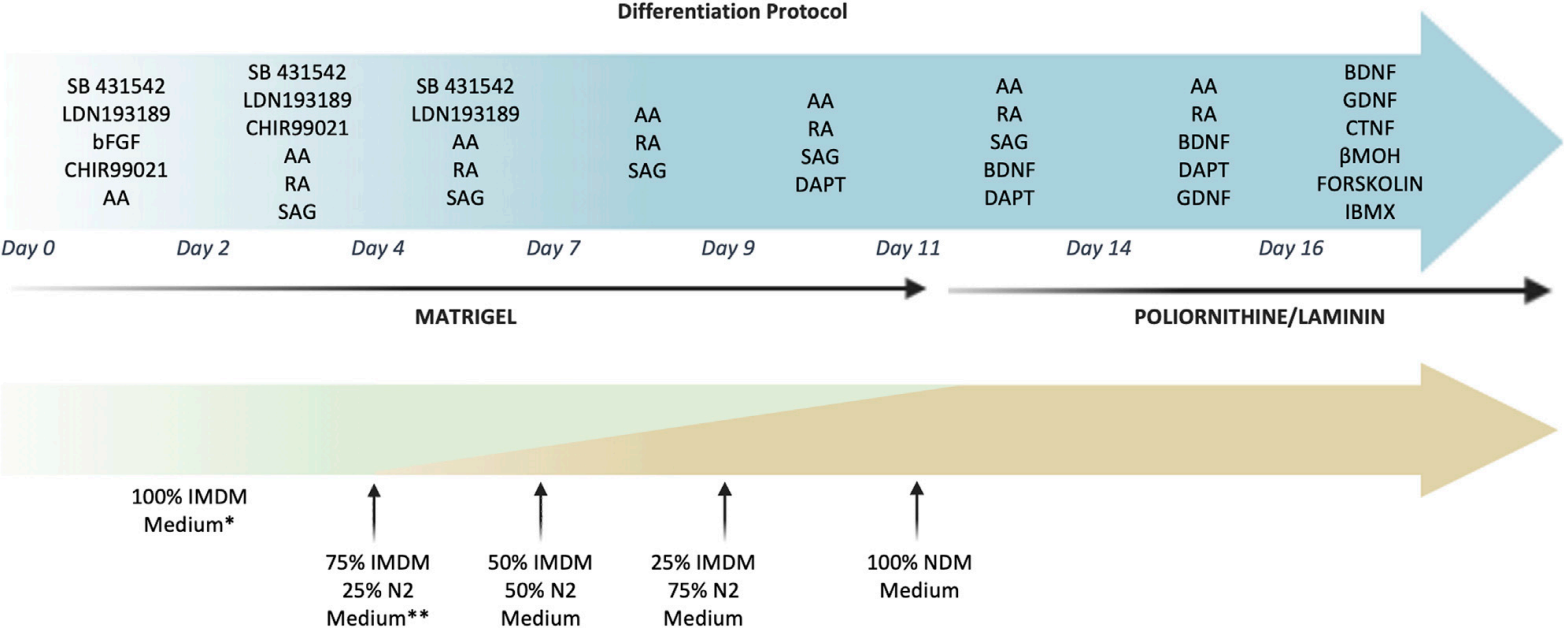
Timeline and culture conditions for the MN differentiation of hAFSCs. The picture reported in detail the small molecules used to induce the MN differentiation. Cells were grown on Matrigel until day 11 and then were detached and replated on polyornitine/laminin plates. During the differentiation process the IMDM medium was gradually replaced with N2 medium until day 11, when it was changed into Neurobasal medium supplemented with B27. *IMDM medium: IMDM 20%FBS supplemented 1% penicillin/streptomycin, 2 mM L-glutamine. **N2 medium: IMDM supplemented with N2, 1% penicillin/streptomycin and 2 mM L-glutamine.

From day 4, the IMDM medium was gradually replaced with increasing concentrations of N2 medium (IMDM, 1% penicillin/streptomycin, 2 mM L-glutamine, N2 supplement 1:100, B27 w/o vitamin A (B27-) 1:50). At day 11, cells were detached and replated (1.3 × 10^5^cell/cm^2^) on polyornithine/laminin coated plates in Neurobasal medium (Thermo Fisher Scientific) supplemented with N2, B27 1:50. (Figure 1).

### Co-cultures of hAFSCs -derived MNs and myotubes

For the co-culture, on day 11 the detached differentiating hAFSCs were seeded on the differentiated C2C12 myotubes (ratio 1:2) in NDM medium supplemented as reported above. When required, hAFSC-derived MNs were loaded with the long-lasting vital dye ViaFluor SE 405 5 µM (Biotium Inc., Fremont, CA, United States) before the plating on the myotubes.

For the non-contacting co-culture, cells were cultured in a µ-slide co-culture dish (Ibidi GmbH, Gräfelfing, Germany).

### RNA extraction and reverse transcription

Cells were lysed with QIAzol lysis reagent (QIAGEN, Germany) and the total RNA was extracted using the miRNeasy Mini Kit (QIAGEN, Germany) according to the manufacturer’s procedure. For reverse transcription, 1 μg of RNA was retrotranscribed by the High-Capacity cDNA reverse transcription kit (Thermo Fisher Scientific) according to the manufacturer’s procedure.

### Real time quantitative PCR

For all the examined mRNAs, qPCR analysis was performed using SYBR green (PowerUp SYBR Green Master mix, Thermo Fisher Scientific MA United States) as previously described (Gaggi et al., 2020a). The run method consisted of the following steps: 95°C for 10 min, 95°C for 15 s, 60°C for 1 min. Steps 2 and 3 were repeated for 40 cycles. The authenticity of the PCR products was verified by melt-curve analysis. Each gene expression value was normalized to 18S. Since some MN-specific genes were undetectable at day 0, the fold changes were expressed in relation to day 11, as previously reported by Shimojo et al. (Shimojo et al., 2015) using the ΔΔCt method. The primers used are listed in Table 1.

**TABLE 1:** qPCR primer sequences.

Gene	Sequence (5′–3′)
Endo-*OCT4*-FW Aasen et al. (2008)	GGG​TTT​TTG​GGA​TTA​AGT​TCT​TCA
Endo-*OCT4*-RV Aasen et al. (2008)	GCCCCCACCCTTTGTGTT
PAX6-FW	CCA​ACT​CCA​TCA​GTT​CCA​ACG
PAX6-RV	GGC​TGC​TAG​TCT​TTC​TCG​GG
HB9-FW Yu et al. (2020)	GCA​CCA​GTT​CAA​GCT​CAA​C
HB9-RV Yu et al. (2020)	GCT​GCG​TTT​CCA​TTT​CAT​CC
ISL1-FW	GCC​TGC​TTT​TCA​GCA​ACT​GG
ISL1-RV	GCC​TCA​ATA​GGA​CTG​GCT​ACC
18S-FW Rubino et al. (2021)	CAT​GGC​CGT​TCT​TAG​TTG​GT
18S-RV Rubino et al. (2021)	CGC​TGA​GCC​AGT​CAG​TGT​AG

### Immunofluorescent analysis

Immunofluorescent analysis was performed as previously described (D’amico et al., 2016; Di Credico et al., 2021a). Briefly, cells were fixed with 4% paraformaldehyde for 10 min, permeabilized with 0.5% Triton X-100 for 15 min and incubated in 5% bovine serum albumin (BSA) for 20 min at room temperature. Cells were subsequently incubated with anti-HB9 Alexa Fluor 488 conjugated 1:50 (Cell Signaling Danvers, MA, United States), anti-NF-L-Alexa Fluor 488 conjugated 1:50 (Cell Signaling Danvers, MA, United States), anti-VaChT 1:100 (Sigma-Aldrich, Saint Louis, MO, United States) followed by the appropriate secondary antibody conjugated with Alexa Alexa Fluor 546 1:100 (Invitrogen, Carlsbad, CA, United States). Nuclei were counterstained with DAPI (Thermo Fisher Scientific, Waltham, MA, United States).

To detect the presence of the neuromuscular junction, hAFSC-derived MNs were stained with the vital staining ViaFluor SE 405 5 µM (Biotium Inc., Fremont, CA, United States), whereas the myotubes were marked with α-bungarotoxin (α-BTX) Alexa Fluor 488 conjugated (Thermo Fisher Scientific, Waltham, MA, United States).

Images were acquired using a confocal system Zeiss LSM800 equipped with an inverted microscope Axio-observer D1 and an objective W-Plan-Apo 40X/1.3 DIC (Carl Zeiss, Jena, Germany). The image analyses were performed by ZEN Software (Carl Zeiss) or Celleste Image Analysis Software (Thermo Fischer Scientific).

### Contraction analysis

The degree of muscle contraction was quantified by the Tracking distance tool of the Celleste Image Analysis Software (Thermo Fisher Scientific): this option allows to automatically select all moving objects contained in the region of interest and tracking their movements over time. The distance covered by each point of the contracting myotubes provides an indirect quantification of the contraction. Numerical data were exported from the data table option and used for statistical analysis. In addition, graphs were obtained to visually represent the myotubes contraction. The distance was expressed as pixel (*y*-axis) over time (*x*-axis). The selected regions of interest were maintained constant to permit the comparison among the different experimental conditions. The video were uploaded in the analysis software as MP4 extension.

### Calcium imaging

Intracellular Ca^2+^ levels were monitored by using the dye Fluo4-acetoxymethyl ester (Fluo4/AM, Thermo Fisher Scientific). An upright microscope (Zeiss Axio Examiner; Carl Zeiss) was used, equipped with 40X 0.75 NA water-immersion objectives connected by optical fiber to a 75 W Xenon lamp and a monochromator (OptoScan; Cairn Instrument, United Kingdom). Sub-millisecond bandpass and wavelength controls were used with a back-illuminated camera (EMCCD, Evolve 512; Photometrics, Tucson, United States). The cells were incubated with 5 µM Fluo-4/AM in normal external solution (NES (in mM: 140 NaCl, 2.8 KCl, 2 CaCl_2_, 2 MgCl_2_, 10 glucose, 10 Hepes, pH 7.3) supplemented with 1% (w/v) bovine serum albumin for 40 min at 37°C. Recordings on Fluo4-loaded cells were performed in NES. The fluorescence was acquired by setting excitation at 488 nm and images acquired at 20 frames/s with an EMCCD camera and stored on an interfaced computer for off-line analysis using Metafluor (Molecular Device, Sunnyvale, CA, United States). The temporal analysis was calculated as the mean fluorescence intensity signal in a selected cell area, as F/F_0_, where F is the fluorescence emission of a single loaded cell acquired during a time lapse, and F_0_ is the mean fluorescence intensity of the same cell calculated from first images acquired.

### Statistical analysis

All data are presented as the mean ± SD. A statistical analysis was performed using the one-way analysis of variance (ANOVA) and Tukey’s post-hoc analysis. The level of significance was set at *p* < 0.05.

## Results

### hAFSC characterization

hAFSCs were characterized by flow cytometry between passages 3 and 4. According to previous data (Di Baldassarre et al., 2018b), they expressed both pluripotent (OCT4, SSEA4, SOX2, NANOG, TRA1-60, C-KIT) and mesenchymal markers CD90 (Table 2).

**TABLE 2:** Phenotypic characterization of hAFSCs.

Markers	Positive cells (%)
Pluripotency Markers	
OCT4	18.5 ± 5.2
SSEA4	65.3 ± 6.1
SOX2	53.0 ± 2.3
NANOG	76.2 ± 3.1
TRA1-60	12.1 ± 2.8
C-KIT	1.0 ± 1.3
Mesenchymal markers	
CD90	73.1 ± 4.1
CD105	68.4 ± 3.8
Hematopoietic markers	
CD34	Negative
CD45	Negative

### hAFSC acquire a MN phenotype during the differentiation process

**FIGURE 2:**
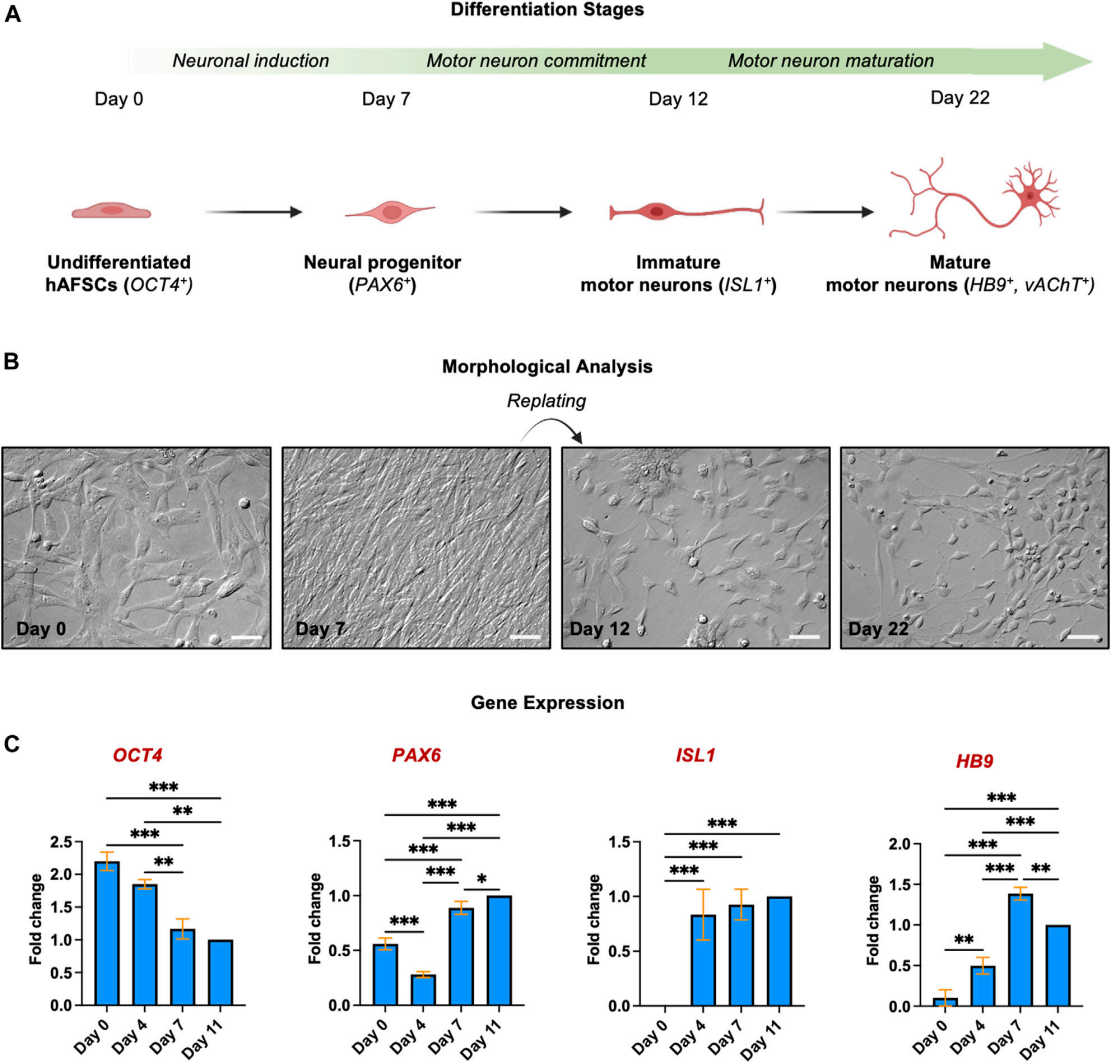
Morphological and gene expression analysis of hAFSC during the differentiation into MNs **(A)** Schematic representation of key points of MN differentiation. **(B)** Morphological analysis of different differentiation steps. Magnification 20x, scale bar 50 µm. Pictures are representative of 3 independent experiments. **(C)** Gene expression of *OCT4, PAX6, ISL1* and *HB9* was quantified in hAFSCs by qPCR at different time points during the differentiation process, as indicated. The fold changes were expressed in relation to day 11. *18S* was used as reference gene. The graphs show the Mean ± SD of 3 independent experiments *p < 0.05.

To elucidate whether hAFSCs can be driven towards the MN fate, cell morphology was monitored during the different phases of the differentiation process (Figure 2): undifferentiated cells, characterized by the typical fibroblastic-like morphology, were sequentially exposed to specific molecules to drive the neural induction (Chambers et al., 2009; Chavali et al., 2020; Galiakberova and Dashinimaev, 2020; Gaggi et al., 2021); these MNs progenitors were then detached, replated and treated with specific factors known to promote the MNs differentiation (Maury et al., 2015): immature MNs showed the typical polygonal cell body with few and short dendrites (Day 12). The final maturation was then obtained thanks to the action of neurotrophic factors and was characterized by the increase of dendrites arborization and cell-cell connections (day 22) (Ishii et al., 1993; Hanson et al., 1998; Maury et al., 2015). The morphological analysis was paralleled by the study of expression of genes involved in the spinal MN differentiation; this transcript analysis was performed until day 11, when the cells were replated either on polyornithine/laminin coated plates or on myotubes (Figure 2). Data showed that, as expected, the pluripotency marker OCT4 gradually decreased, whereas *PAX6, ISL1* and *HB9* that represent early and late MN markers, were upregulated starting rising already after 4 days of the differentiation process.

**FIGURE 3:**
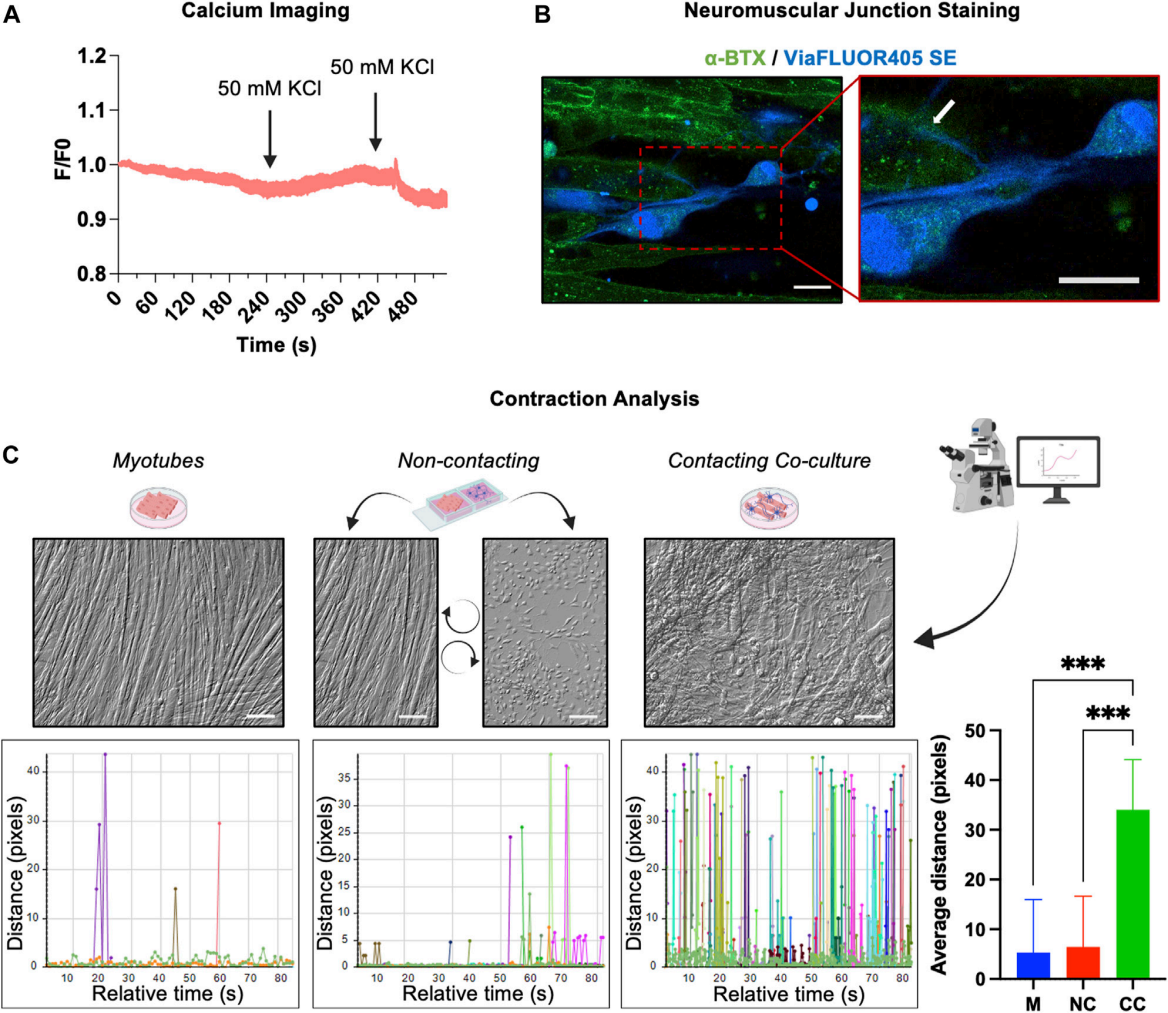
Immunofluorescent analysis of late MN markers. Immunostaining and the relative fluorescence intensity for **(A)** NF-L (green), **(B)** vAChT (red) and **(C)** HB9 (green) in undifferentiated cells (day 0) and hAFSC-derived MNs (day 22). The nuclei were counterstained with DAPI. Arrows indicate the cytoskeleton localization of NF-L in **(A)**, the vesicular staining pattern of vAChT in **(B)** and the HB9 perinuclear localization in **(C)**. Original magnification: 40x, scale bar 20 μm. Red rectangle represents an enlarged area: magnification 60x. Immunofluorescence images are representative of 3 independent experiments.

To confirm the acquisition of the MN phenotype, the expression of proteins involved in the consolidation of MN identity was checked immunocytochemically. As the MN maturation was morphologically evidenced by the increased number, size, and complexity of neurites after 3 weeks of culture, and since stem cells-derived MNs are generally considered mature after 21 days of differentiation (Bianchi et al., 2018), we analyzed the protein expression of the late MN markers HB9, Vesicular acetylcholine transporter (vAChT) and Neurofilament L (NF-L) at day 22. Immunofluorescence revealed that all these markers, undetectable in undifferentiated cells, were strongly and homogeneously expressed by the hAFSC-derived MNs (Figure 3). In particular, NF-L, which is involved in the formation of neuronal cytoskeleton, organized in cytoplasmatic tubular structures, which resembles the intermediate filaments; vAChT, that is a unique marker for cholinergic neurons, evidenced a typical vesicular staining pattern both in the cell body and along the axon; finally, HB9, the spinal MN specific transcription factor, was characterized by a diffuse intracellular distribution both in the nuclei and in the cytoplasm, showing intense fluorescence in the perinuclear region, where the Golgi complex and the endoplasmic reticulum complex are located.

### hAFSC derived MN like cells established a functional neuromuscular junction with myotubes

**FIGURE 4:**
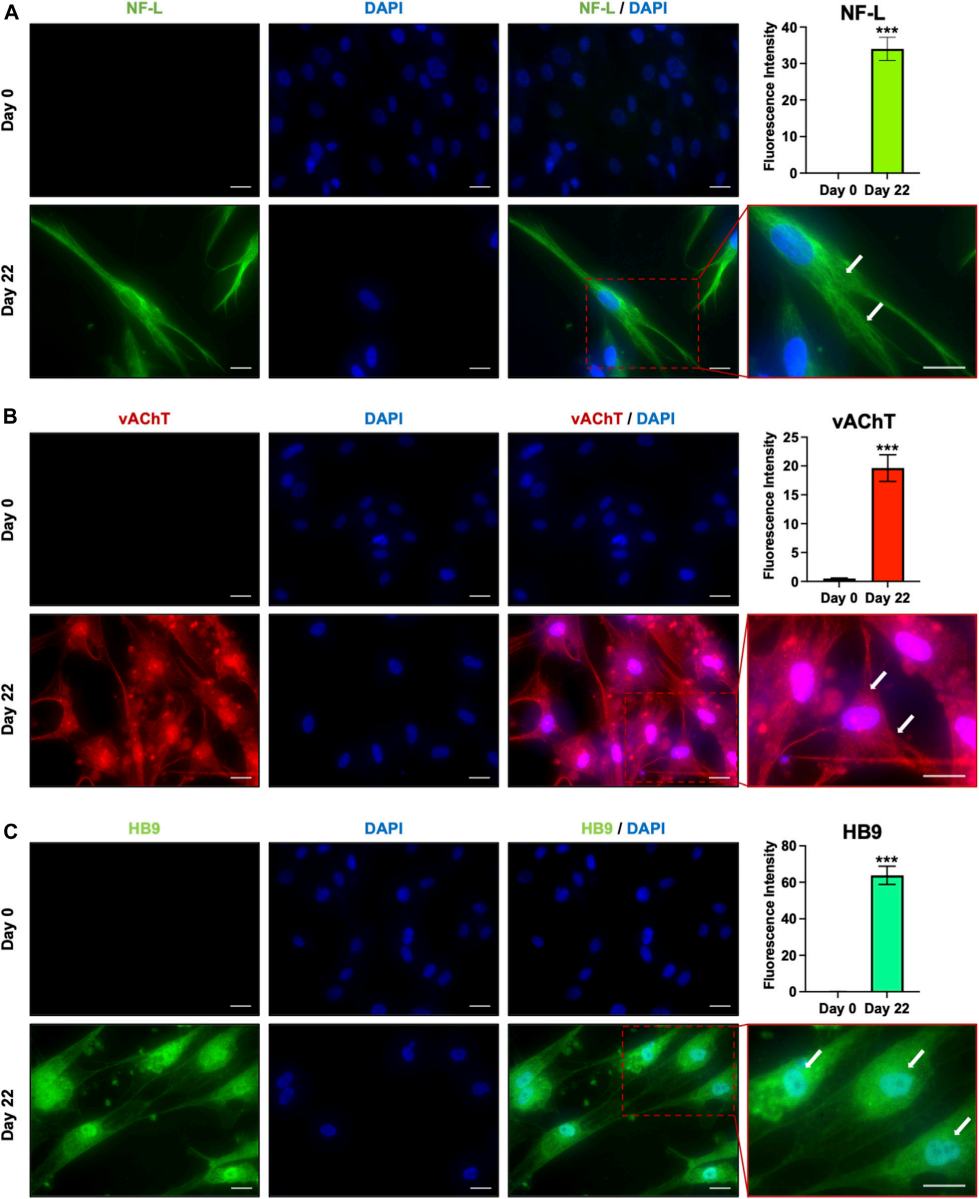
Functional features of hAFSC-derived MNs. **(A)** Calcium imaging in hAFSC-derived MNs after 22 days of differentiation. Cells were stimulated with 50 mM of KCl. F/F0 represents the ratio of fluorescence intensity for each cell at the indicated time. **(B)** NMJ detection in the co-culture of hAFSC-derived MNs and myotubes. MNs were labelled with a blue vital staining, whereas the AChRs on myotubes were stained with the α-BTX (green). Red rectangle represents enlarged area (60x). White arrow indicates neuronal terminals ending on the α-BTX (AChRs). Magnification 40x, scale bar 20 µm. The picture is representative of 3 different experiments. **(C)** Video analysis of the muscle cell contractions in single culture of myotubes, in a contacting co-culture (hAFSC-derived MNs plus myotube) and in a non-contacting co-culture between hAFSC-derived MNs and myotubes. The movements of different points were tracked and quantified by Celleste Image Analysis Software. Graphs are representative of the distance covered by moving points inside three differently ROI.

After the phenotypic characterization, we verified whether after 3 weeks of differentiation, the morphological maturation of the hAFSC-derived MNs was accompanied by the acquisition of some neural functional properties. Using fluorescence video imaging, the cells were stimulated with 50 mM KCl, a depolarizing agent that allows the calcium entry into the cells by the activation of the L-type Ca^2+^ channels present on the sarcolemma. Data obtained from calcium imaging showed that the differentiated cells did not respond to KCl stimulation, suggesting that after day 22, the hAFSC-derived MN like cells were still functionally immature (Figure 4A).

However, since the functional output of MNs is the muscle cells contraction, the ability of hAFSC-derived-MNs to form neuromuscular junctions (NMJ) with myotubes was checked. For this purpose, after the first 11 days of culture, differentiating hAFSC were detached, loaded with a long-lasting blu vital dye, and then seeded on differentiated C2C12 myotubes. After further 10 days of co-culture, the acetylcholine receptors (AChRs) present on the myotubes were marked with a fluorescent α-bungarotoxin (α-BTX) and the samples analysed. Immunofluorescence evidenced neurites ending on the AChRs of the myotubes (Figure 4B), thus suggesting that hAFSC-derived MNs were competent in developing NMJ. The activity of these NMJ was finally proved by the observation that, while no or very weak contractile activity was observed in C2C12 culture alone, myotubes strongly contract when cultured in presence of hAFSC-derived MNs (Supplementary Videos 1, 2). The degree of the C2C12 myotube contractions was measured by analysing the cell moving on the recorded videos: while in absence of hAFSC-derived MNs only small movements were registered, in the co-culture in which myotubes were in contact with the hAFSC-derived MNs, the cell track evidenced significant shifts of the myotubes at high frequency.

To rule out the possibility that the contractile activity observed in the co-cultures was due to a crosstalk between the cell types via secretory pathways, we set up a non-contacting co-culture, in which hAFSC-derived MNs and C2C12 myotubes grew separately sharing the same media and soluble factors. No contraction movements were observed microscopically in these non-contacting co-culture (Figure 4C).

These results proved that hAFSC-derived MNs can integrate with myotube system creating functional NMJ.

## Discussion

The use of the stem cells is a promising strategy for the treatment of the neurodegenerative disorders, including MNDs. In addition, thanks to their capacity to differentiate into the different neuron types, they may represent a very valuable *in vitro* model to investigate the molecular bases of NMDs, thus contributing to the development of new and more effective treatments. To date, terminally differentiated MNs can be obtained from hESCs and hiPSCs. The use of these two cellular models ensure high yield of MNs, but it has also some significant disadvantages: hESCs rises important ethical issues, whereas the genetic instability and the epigenetic memory of hiPSCs still cast shadows on their clinical applications (Koch et al., 2009; Mattis and Svendsen, 2011); all these barriers have hampered the use of hESCs and hiPSCs in the medical context.

Perinatal stem cells can be easily obtainable in an inexpensive and non-invasive manner from the amniotic fluid, placenta, and umbilical cord; deriving from fetal annexes, these stem cells lack classical MHC class II antigens and this low immunogenicity give them the potential to escape recognition by alloreactive CD4^+^ T cells (Gaggi et al., 2019). Mid-trimester human amniotic fluid cells are a heterogeneous population that includes stem cells with intermediate phenotype between the pluripotent and the adult stem cells: indeed, they are characterized by a high proliferation rate, the expression of both pluripotent and mesenchymal markers while maintaining the non-tumor-forming properties of adult cells. All these characteristics encourage the study of their use in regenerative medicine (Gaggi et al., 2020c). As Amniotic Fluids contain a heterogeneous mixture of cell types, the phenotyping of the amniotic cell samples is essential to characterize the starting populations in differentiation experiments. We previously demonstrated that only samples expressing pluripotent and multipotent stem markers such as SSEA4, OCT4 and CD90 may form Embryo Bodies that provide a suitable microenvironment for the differentiation of the residing cells (Antonucci et al., 2014; Di Baldassarre et al., 2018b).

Most of the studies on the differentiation potential of hAFSCs have been carried out by isolating c-Kit^+^ cells from the initial heterogeneous population. However, data on the surface markers of hAFSCs are not consistent among the different studies and it is still unclear whether sorting for c-Kit expression is necessary or appropriate. Arnhold and coll (Arnhold et al., 2011) evidenced that the c-Kit^+^ cell fractions preferentially differentiate into mesodermal lineages, while the unsorted population is more prone to the neuronal commitment. This opportunity to develop *in vitro* neuronal potential without recurring to any immunoselective method is crucial as the absence of xenogenic antibodies is essential in advancing cell therapy applications (Vemuri et al., 2007).

To date, data on the capacity of hAFSCs to differentiate into neurons are often contradictory. Some groups reported that hAFSCs can express the neuron specific class III β-tubulin (Tuji) when stimulated with βMOH and bFGF. Maraldi et al. demonstrated that c-KIT^+^ hAFSCs expressed neuronal and glial markers, such as Glial fibrillary acidic protein (GFAP), Tuji, Synuclein (SYN) and S-100 when treated with RA (Maraldi et al., 2014). Anyway, no terminal differentiation into a specific class of neurons has been reported yet. Here we reported for the first time that, adapting protocols (Maury et al., 2015; Shimojo et al., 2015; Bianchi et al., 2018) that mimics the different steps of MNs differentiation *in vivo*, hAFSCs can differentiate into mature MNs. The neural induction was obtained modulating the SHH and the RA signaling, together with Wnt activation: in this phase, the generation of MN progenitors was proved by the expression of Pax6, one of the earliest MN markers. The MN commitment and maturation was then guided by the modulation of the Notch pathway by DAPT, that determines an increased yield of ISL1^+^ and HB9^+^ cells (Maury et al., 2015). *In vivo*, *Isl1* and *Hb9* are among the first genes expressed in post-mitotic spinal MNs. These two transcription factors play a pivotal role in the consolidation of MN identity, as when their expression is reduced, the developing MNs transdifferentiate into interneurons (Kim et al., 2016), while their ectopic overexpression is sufficient to trigger the MN differentiation (Arber et al., 1999). ISL1 and HB9 continue to be expressed also in mature MNs and they are the most used marker to detect the presence of terminally differentiated MNs (Trawczynski et al., 2019). We found that hAFSC-derived MNs express HB9, whose significant nuclear localization suggests its transcriptional activity, as expected in differentiated cells (Leotta et al., 2014); moreover, HB9 marked also the perinuclear area of the endoplasmic reticulum and the Golgi complex: as these subcellular compartments are closely linked not only by their location in the cytoplasm, but also in their function in the newly synthesized proteins, the HB9 localization in the perinuclear compartment suggests a still active synthesis of the transcription factor in maturing hAFSC-derived MNs. The MNs identity was also supported by the NF-L and vAChT expression: NF-L, that contributes to the structural support for the highly asymmetric geometries of neurons and for the marked radial expansion of the axons (Varhaug et al., 2019), organized relatively sparse and tortuous in dendrites and perikarya forming tubular structures resembling cytoskeleton filaments, while vAChT that provides the storage of acetylcholine into vesicles for its transportation along the neurites (Ferguson et al., 2003; de Castro et al., 2009), localized mainly along the axons and in cell body depicting a typical granular pattern (Ichikawa et al., 1997).

Our data also demonstrated that hAFSC-derived MN can integrate with myotubes forming physiologically relevant connections, as evidenced by the muscular contractile activity observed after about 10 days of co-culture: this timing is the same reported for the NMJ generation from hiPSCs-derived MNs (Puttonen et al., 2015; Yi et al., 2018). We demonstrated immunocytochemically that hAFSC-derived MNs terminate with their axons on the sarcolemma at the AChRs level. It is well known that the AChRs typically gather beneath the motor nerve axon, but in our co-culture system they were more spread along the sarcolemma. This is probably due the immaturity of the C2C12 myotubes: indeed, nicotinic AChRs are initially dispersed throughout the membrane, and only over time they concentrate on the postsynaptic fiber following the neuronal signals (Zhang and Peng, 2011). However, even if the synaptic maturation was not complete, the functionality of the NMJ was proved by the robust contractile activity of the C2C12 myotubes. This observation seems in contrast with analysis of the calcium transients performed on the single cultures of hAFSC-derived MNs after 3 weeks of culture, that evidenced only a slow oscillatory pattern and a non-responsiveness to the depolarizing agent KCl. The discrepancy between this electrophysiological immaturity of hAFSC-derived MNs cultured alone on one hand and the competence to generate functional NMJ on the other can be attributed to the significant contribution of skeletal muscle cells to neuronal physiology: it is well known, indeed, that at the NMJ levels, there is bidirectional crosstalk between MNs and muscle cells. In this scenario, the hypothesis that the myotubes may produce some molecules, such as neurotropic factors, cytokines, exosomes or microparticles (Matthews et al., 2009; Di Credico et al., 2020; Di Credico et al., 2021b; Saini et al., 2021), that might increase the support the maturation of hAFSC-derived MNs cannot be ruled out; however, further investigation on the MNs and myotube secretome, cultured alone or together, are required to specifically address this point.

In conclusions, we demonstrated for the first time that hAFSCs can overcome their mesenchymal restriction and give rise to an ectodermal derivate generating MNs like cells: being easily obtainable and safely transplantable for their low immunogenicity, hAFSCs may represent a promising and useful tool in the study and treatment of the MNDs.

## Data Availability

The original contributions presented in the study are included in the article/Supplementary Material, further inquiries can be directed to the corresponding author.

## Supplementary material

Supplementary Video S1 Myotubes cultured alone

Supplementary Video S2 Contacting co-culture between hAFSC-derived MN and myotubes
